# IL-23-induced macrophage polarization and its pathological roles in mice with imiquimod-induced psoriasis

**DOI:** 10.1007/s13238-018-0505-z

**Published:** 2018-03-05

**Authors:** Yuzhu Hou, Linnan Zhu, Hongling Tian, Hai-Xi Sun, Ruoyu Wang, Lianfeng Zhang, Yong Zhao

**Affiliations:** 1 State Key Laboratory of Membrane Biology, Institute of Zoology, Chinese Academy of Sciences, University of Chinese Academy of Sciences, Beijing, 100101 China; 2 Department of Oncology, The Affiliated Zhongshan Hospital of Dalian University, Dalian, 116001 China; 3 Key Laboratory of Human Diseases Comparative Medicine, Ministry of Health, Institute of Laboratory Animal Science, Chinese Academy of Medical Sciences and Peking Union Medical College, Beijing, 100021 China

**Keywords:** interferon-gamma, interleukin-17, interleukin-23, imiquimod-induced psoriasis, macrophage polarization

## Abstract

Macrophages acquire distinct phenotypes during tissue stress and inflammatory responses. Macrophages are roughly categorized into two different subsets named inflammatory M1 and anti-inflammatory M2 macrophages. We herein identified a unique pathogenic macrophage subpopulation driven by IL-23 with a distinct gene expression profile including defined types of cytokines. The freshly isolated resting mouse peritoneal macrophages were stimulated with different cytokines *in vitro*, the expression of cytokines and chemokines were detected by microarray, real-time PCR, ELISA and multiple colors flow cytometry. Adoptive transfer of macrophages and imiquimod-induced psoriasis mice were used. In contrast to M1- and M2-polarized macrophages, IL-23-treated macrophages produce large amounts of IL-17A, IL-22 and IFN-γ. Biochemical and molecular studies showed that IL-23 induces IL-17A expression in macrophages through the signal transducer and activator of transcription 3 (STAT3)-retinoid related orphan receptor-γ T (RORγT) pathway. T-bet mediates the IFN-γ production in IL-23-treated macrophages. Importantly, IL-23-treated macrophages significantly promote the dermatitis pathogenesis in a psoriasis-like mouse model. IL-23-treated resting macrophages express a distinctive gene expression prolife compared with M1 and M2 macrophages. The identification of IL-23-induced macrophage polarization may help us to understand the contribution of macrophage subpopulation in Th17-cytokines-related pathogenesis.

**Electronic supplementary material:**

The online version of this article (10.1007/s13238-018-0505-z) contains supplementary material, which is available to authorized users.

## Introduction

Macrophages demonstrate significant plasticity and are able to modify their phenotype and function in response to the surrounding microenvironments (Murray and Wynn, 2011). It is well known that macrophage polarization display tremendous heterogeneity and is involved in tissue remodeling and pathogenesis. Recently, an elegant study evaluated the transcriptome of human macrophages induced by a variety of stimuli and revealed an extraordinary spectrum of macrophage activation states that far extend the current M1 versus M2-polarization model (Xue et al., 2014). Importantly, the diverse macrophage subsets can have drastic effects on health and disease within the tissues where they reside (Labonte et al., 2014).

IL-23, one member of the IL-12 cytokine family, is crucial in the pathogenesis of psoriasis, experimental autoimmune encephalomyelitis (EAE), collagen-induced arthritis (CIA), inflammatory bowel disease (IBD) (Tonel et al., 2010; Teng et al., 2015) and leukocyte adhesion deficiency type 1 (LAD1) (Moutsopoulos et al., 2017). Polymorphisms in the gene encoding the IL-23 receptor (IL-23R) are important susceptibility factors for Behcet’s disease, ankylosing spondylitis, and IBDs like Crohn’s disease and ulcerative colitis (Remmers et al., 2010; Kadi et al., 2013). It is known that IL-23 is essential for the terminal differentiation of IL-17-producing T effector cells (Park et al., 2005; McGeachy et al., 2009), which were initially shown to be a chief pathogenic cell population in EAE and CIA (Duerr et al., 2006; Remmers et al., 2010), human psoriasis (Wilson et al., 2007; Lubberts, 2015) and LAD1 (Moutsopoulos et al., 2017). However, in addition to acting on Th17 cells, IL-23 also regulates the function of innate lymphocytes (Guo et al., 2012). IL-23R is predominantly found on activated memory T cells, natural killer (NK) cells, and innate lymphoid cells (ILCs), and at lower levels on monocytes, macrophages, and dendritic cells (DCs) in humans; whereas mouse IL-23R is expressed on activated T cells, ILCs, γδ T cells, macrophages and DCs (Kastelein et al., 2007; Awasthi et al., 2009; Aychek et al., 2015). Importantly, studies have demonstrated that IL-23 induces these innate cells to secrete IL-17 and/or IL-22, although it remains unknown whether IL-23 affects the functional development of IL-23R-expressing innate cells *in vivo* (Cella et al., 2009; Guo et al., 2012; Paget et al., 2012).

We herein demonstrate that IL-23-treated monocyte/macrophages selectively produce IL-17A, IL-22 and IFN-γ, and display a distinct lineage gene expression profile in sharply contrast to M1 and M2 subsets. Importantly, M(IL-23) macrophages significantly promote the severity of dermatitis pathogenesis in a mouse psoriasis-like model. Thus, our findings reveal a previously unappreciated macrophage polarization driven by IL-23 with unique cell surface markers and cytokine-producing gene profile.

## Results

### IL-23 induces a distinct macrophage gene expression profile

**Figure 1:**
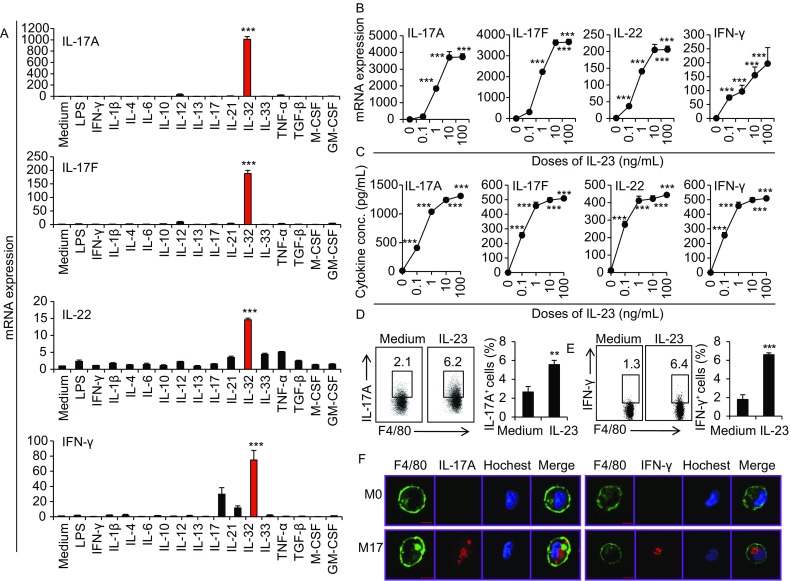
**Cytokine expression of macrophages stimulated with different cytokines**. (A) The freshly isolated peritoneal macrophages (PEMs) were stimulated with different cytokines and LPS for 48 h. Cytokine mRNA expression were detected by real-time PCR. (B) mRNA expression of IL-17A, IL-17F, IL-22 and IFN-γ in PEMs treated with different doses of IL-23. (C) Concentrations of IL-17A, IL-17F, IL-22 and IFN-γ in the media of PEMs after IL-23 treatment for 48 h. (D) The percentages of IL-17A^+^ cells in F4/80^+^ PEMs were detected by a flow cytometry. (E) The percentages of IFN-γ^+^ cells in F4/80^+^ PEMs treated with IL-23. (F) The expression of IL-17A and IFN-γ in PEMs were determined using two-photon microscope. Data were shown as mean ± SD (*n* = 3). **P < 0.01, ***P < 0.001 compared with the control

To explore the roles of different cytokines on the expression of IL-17 family members include IL-17A, IL-17B, IL-17C, IL-17D, IL-17E (also called IL-25) and IL-17F in macrophages, we firstly detected the expression of these genes in freshly isolated mouse peritoneal resident macrophages after different cytokines and LPS stimulation for 48 h by real-time PCR. Among the 15 cytokines and LPS studied, only IL-23 significantly promoted IL-17A and IL-17F expression, while resting macrophages expressed almost undetectable levels of IL-17A and IL-17F (*P* < 0.001, Fig. 1A). In addition, IL-23 also significantly induced IL-22 and IFN-γ expression in a specific manner compared to other cytokines and LPS stimulation (*P* < 0.001, Fig. 1A). IL-23 induced the mRNA and protein expression of Th17-type cytokines in dose- and time-dependent manners as determined by real-time PCR and ELISA assays (Figs. 1B, 1C and S1). The expressions of IL-17A and IFN-γ in IL-23-treated macrophages were further confirmed by flow cytometry and confocal microscopy staining (Fig. 1D–F). The low percentage of IL-17A^+^ macrophages might be due to the limited IL-23R expression on CD11b^+^F4/80^+^ macrophages (Fig. S2). However, IL-23 failed to induce significantly high levels of IL-17C, IL-17D and IL-17E expression in macrophages (Fig. S3). Furthermore, IL-23 significantly induced IL-17A, IL-17F, IL-22 and IFN-γ expression in tissue resident macrophages isolated from spleens, lungs and liver as well (Fig. S4). To exclude the potential contamination of other immune cells like T cells, B cells and ILCs during the differentiation process, we sorted the peritoneal resident cells of naïve mice to obtain highly purified F4/80^+^ cells. It is true that more than 99% of the sorted cells were CD11b^+^F4/80^+^ cells and indeed these cells also expressed high levels of IL-17A and IL-17F after IL-23 treatment for 48 h (Fig. S5). In addition, the *in vitro* bone marrow-derived macrophages also expressed higher levels of IL-17A, IL-17F, IL-22 and IFN-γ after IL-23 stimulation (Fig. S6). Thus, IL-23 promotes IL-17A, IL-17F, IL-22 and IFN-γ expression in resting mouse macrophages in a specific manner. However, the IL-23-induced expression of IL-17A, IL-17F, and IL-22 in macrophages were significantly lower than Th17 cells (Fig. S7A), as well as the lower IFN-γ expression when comparing with Th1 (Fig. S7B).

**Figure 2:**
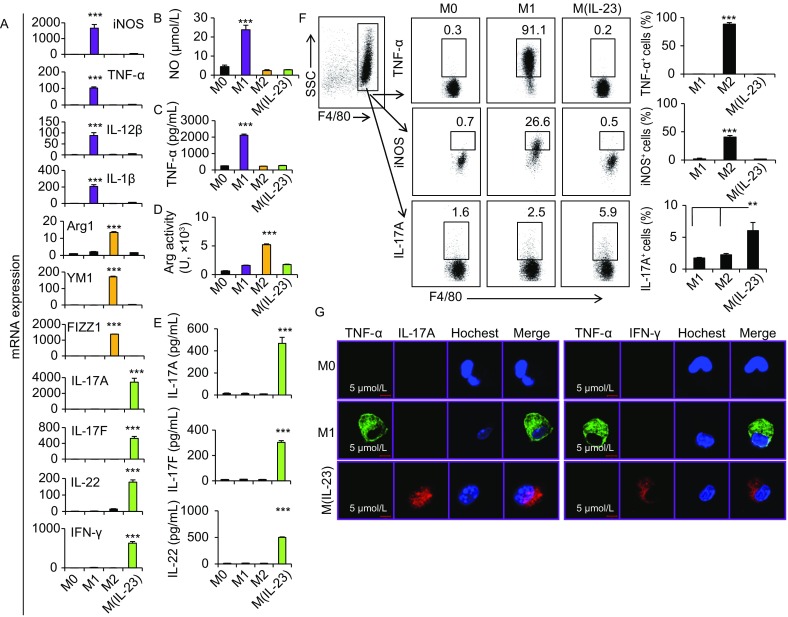
**IL-23-treated macrophages produce a distinctive panel of cytokines**. (A) The mRNA expression of M1 and M2 marker molecules and Th17-cytokines in macrophages were detected by real-time PCR. (B) NO concentrations in M0, M1, M2 and M(IL-23) macrophage culture medium. (C) TNF-α concentrations in the culture medium. (D) Arginase activity of M0, M1, M2 and M(IL-23) macrophages was assayed. (E) Concentrations of IL-17A, IL-17F and IL-22 in the culture medium. (F) Representative flow cytometry results (left) and summarized percentages (right) of TNF-α^+^, iNOS^+^ and IL-17A^+^ cells in the F4/80^+^ macrophages. (G) The expression of TNF-α, IL-17A and IFN-γ in M0, M1 and M(IL-23) were determined using a two-photon microscope. Data were shown as mean ± SD (*n* = 4). **P < 0.01, ***P < 0.001 compared with the control

**Figure 3:**
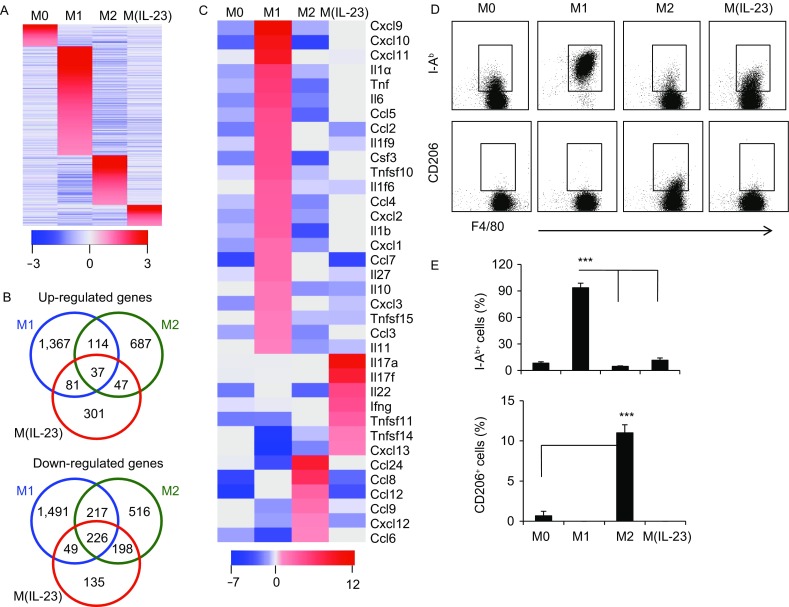
**IL-23-treated macrophages display a distinctive gene expression profile**. (A) Heatmap diagram summarizing gene expression in M0, M1, M2 and M(IL-23) cells. Relative gene expression was depicted according to the color scale shown below the cluster. (B) Venn diagram of up-regulated (top) and down-regulated (bottom) genes in M1, M2 and M(IL-23) compared with M0 cells. (C) Heatmap diagram summarizing cytokines and chemokines expression in M0, M1, M2 and M(IL-23) macrophages. Relative gene expression was depicted according to the color scale. (D) Representative staining of I-A^b^, and CD206 on M0, M1, M2 and M(IL-23) cells. (E) Percentages of I-A^b+^, and CD206^+^ cells in F4/80^+^ M0, M1 and M(IL-23) cells. Data were shown as mean ± SD (*n* = 3). ***P < 0.001 for comparisons between the indicated groups

To investigate whether IL-23 induces a unique macrophage polarization in contrast to M1 and M2 subpopulations, we compared the expression patterns of the subpopulation-related marker genes in macrophages treated with LPS + IFN-γ, IL-4 and IL-23, respectively. Surprisingly, IL-23 failed to induce either the expression of M1 marker genes like iNOS, TNF-α, IL-12 and IL-1β, or M2-related genes like Arg1, YM1 and FIZZ1 (Murray and Wynn, 2011), as detected by quantitative PCR, ELISA and bioactivity assays (Fig. 2A–D), whereas IL-23-treated macrophages specifically expressed IL-17A, IL-17F, IL-22 and IFN-γ (Fig. 2A–E). The distinct expression patterns of TNF-α, iNOS, IFN-γ, IL-17 and IL-22 in M1 and M(IL-23) macrophages were further determined by flow cytometry and confocal assays (Fig. 2F and 2G). To further demonstrate whether M(IL-23) macrophages represent a distinct polarization of macrophages, we thus determined the gene expression profiles of M1, M2 and M(IL-23) cells by microarray analysis. Indeed, M(IL-23) macrophages expressed a unique panel of genes in sharply contrast to M1 and M2 macrophages (Fig. 3A). The microarray data were submitted to the NCBI Gene Expression Omnibus (GEO, http://www.ncbi.nlm.nih.gov/geo, under accession number GSE 102274). M1, M2 and M(IL-23) cells expressed significant different gene profiles with up-regulated 301 genes and down-regulated 135 genes specifically in M(IL-23) cells compared with M1 and M2 cells (Fig. 3B). Interestingly, M1, M2 and M(IL-23) cells expressed distinctive gene profiles of cytokines and chemokines, as determined by mRNA microarray and real-time PCR methods (Figs. 3C and S8). We also found that M1 macrophages expressed high levels of I-A^b^ and M2 macrophages expressed high CD206 on the surface as reported previously (Sun et al., 2012), but M(IL-23) macrophages expressed low levels of I-A^b^ and CD206 molecules (Fig. 3D and 3E). Thus, IL-23-treated macrophages express different cytokines and cell surface markers as M1 and M2 macrophages.

### M(IL-23), M1 and M2 polarizations are reciprocally regulated

**Figure 4:**
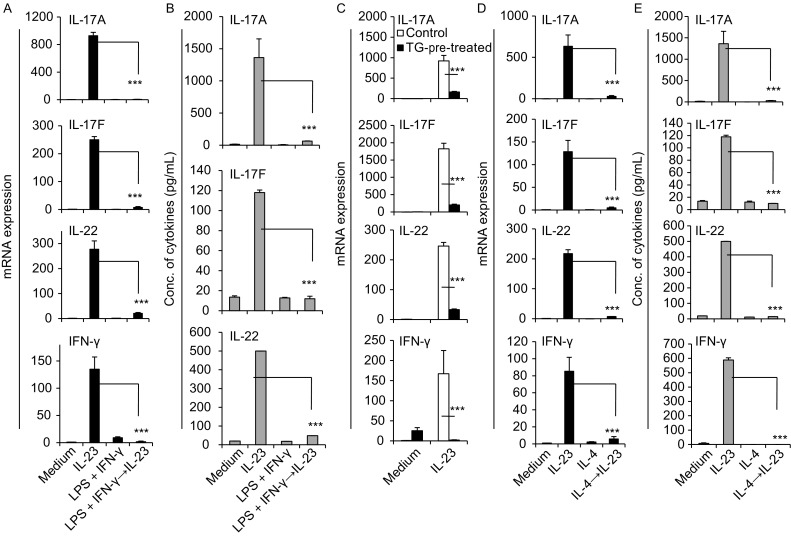
**M(IL-23), M1 and M2 polarizations are reciprocally regulated**. (A) The mRNA expression of IL-17A, IL-17F, IL-22 and IFN-γ in M0, M1, M(IL-23) and IL-23-treated M1 cells. (B) Concentrations of IL-17A, IL-17F and IL-22 cytokines in culture media of M0, M1, M(IL-23) and IL-23-treated M1 cells. (C) mRNA expression of IL-17A, IL-17F, IL-22 and IFN-γ in resting and TG-recruited peritoneal macrophages treated with or without IL-23 for 48 h. (D) mRNA expression of IL-17A, IL-17F, IL-22 and IFN-γ in M0, M2, M(IL-23) and IL-23-treated M2 cells. (E) Concentrations of IL-17A, IL-17F and IL-22 in the culture media of M0, M2, M(IL-23) and IL-23-treated M2 cells. Data were shown as mean ± SD (*n* = 3). ***P < 0.001 for comparisons between the indicated groups

To address whether M(IL-23) macrophages could arise from M1 or M2 cells, we examined the potential differentiation ability into M(IL-23) macrophages from resting M0, M1 and M2 macrophages, respectively. When M1 macrophage polarization was induced from resting macrophages by LPS + IFN-γ as reported (Zhu et al., 2014), these M1-polarized macrophages were remarkably resistant to M(IL-23) induction as indicated by the significantly poor IL-17A, IL-17F, IL-22 and IFN-γ expression when they were subsequently stimulated with IL-23 as determined by real-time PCR and ELISA (*P* < 0.001, Fig. 4A and 4B). Identical results were also observed when M1-polarized peritoneal macrophages freshly isolated from TG-pre-treated mice were used instead of the *in vitro* LPS + IFN-γ-induced macrophages (Fig. 4C). M2 macrophages induced by IL-4 were hard to respond to the subsequent IL-23 treatment in terms of IL-17A, IL-17F, IL-22 and IFN-γ expression (*P* < 0.001, Fig. 4D and 4E). Thus, resting macrophages are susceptible to IL-23-driven M(IL-23) polarization but M1 and M2 macrophages are highly resistant to trans-differentiation into M(IL-23) macrophages.

### The involvement of STAT3-RORγT and T-bet in M(IL-23) polarization

**Figure 5:**
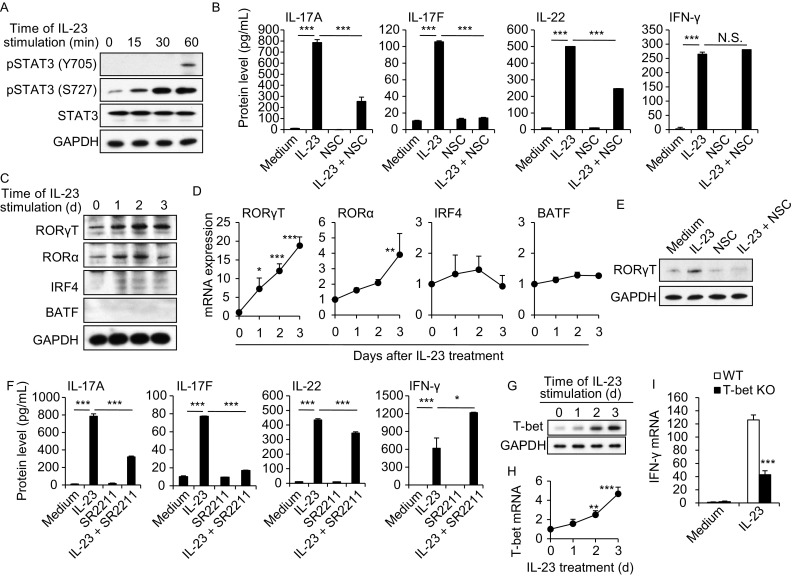
**The involvement of STAT3-RORγT and T-bet in M(IL-23) polarization**. (A) The phosphorylation state of STAT3 in PEMs. (B) The protein concentrations of IL-17A, IL-17F, IL-22 and IFN-γ in culture media of macrophages treated with IL-23 and/or STAT3 inhibitor NSC74859. (C) The expression of RORγT, RORα, IRF4 and BATF in macrophages. (D) The mRNA expression of RORγT, RORα, IRF4 and BATF in macrophages. (E) RORγT expression in macrophages treated with or without IL-23 and/or NSC74859. (F) Protein concentrations of IL-17A, IL-17F, IL-22 and IFN-γ in culture media of macrophages. T-bet protein (G) and mRNA (H) expression in macrophages treated with IL-23. (I) The decreased expression of IFN-γ in T-bet-deficient macrophages. Data were shown as mean ± SD (*n* = 3). *P < 0.05, ***P < 0.001

In order to investigate the signaling pathways activated by IL-23 in macrophages, we performed the pathway analysis based on the gene expression data. As expected, IL-17 and JAK-STAT signaling pathways were activated in macrophages after IL-23 stimulation (Fig. S9A and S9B). Furthermore, IL-23 promoted STAT3 activation in macrophages in terms of the enhanced levels of p-STAT3 (Y705 and S727, Fig. 5A) as previously observed in T-cells (Cho et al., 2006; Teng et al., 2015). Inhibition of STAT3 activation by STAT3-specific inhibitor NSC (NSC74859) significantly decreased the IL-17A, IL-17F and IL-22 mRNA and protein expression in M(IL-23) macrophages, while inhibition of STAT3 failed to inhibit IFN-γ expression (Figs. 5B and S10A). It is reported that RORγT, RORα, IRF4 and BATF are critical transcription factors for Th17 cell induction (Huber et al., 2008; Chung et al., 2009; Ciofani et al., 2012). The expression of RORγT and RORα were enhanced in M(IL-23) macrophages at both mRNA and protein levels (Fig. 5C and 5D), which was further confirmed by confocol imaging analysis (Fig. S11A and S11B). However, no detectable IRF4 and BATF expression in M(IL-23) macrophages was observed in contrast to Th17 cells, as determined by real-time PCR, Western blots and flow cytometry (Figs. 5C, 5D and S12). Thus, the enhanced expression of RORγT and RORα raised the possibility that they are likely involved in IL-23-driven M(IL-23) polarization. Consistently, specific inhibition of RORγT by a chemical SR2211 (Kumar et al., 2012) significantly decreased the IL-17A, IL-17F and IL-22 but not IFN-γ expression in mRNA (*P* < 0.001, Fig. S10B) and protein levels (*P* < 0.001, Fig. 5F). To further confirm the roles of RORγT in the Th17-type cytokines expression in macrophages induced by IL-23, we freshly isolated CD11b^+^F4/80^+^ peritoneal macrophages from RORγT KO and wild-type control mice and then treated these cells with IL-23 *in vitro*. As shown in Fig. S13, significantly less IL-17A, IL-17F and IL-22 expression but not IFN-γ expression was detected in RORγT-deficient macrophages compared with wild-type control macrophages (*P* < 0.01, Fig. S13). As expected, inhibiting STAT3 activity significantly blocked the IL-23-induced RORγT expression in macrophages (Fig. 5E), indicating RORγT is a down-stream molecule in IL-23-activated STAT3 pathway. Thus, IL-23 induces IL-17A, IL-17F and IL-22 expression in macrophages through a STAT3-RORγT-dependent pathway.

To understand the intracellular signal pathway for IFN-γ production in M(IL-23) macrophages, we detected the Th1-related key transcription factor T-bet (Robinson and O’Garra, 2002). The expression of T-bet was significantly up-regulated in macrophages after IL-23 treatment as determined by real-time PCR and Western blots (Fig. 5G and 5H). Macrophages isolated from T-bet KO mice expressed significantly lower IFN-γ after IL-23 treatment (*P* < 0.001, Fig. 5I). However, the T-bet deficiency failed to impact the IL-17A, IL-17F and IL-22 expression in macrophages induced by IL-23 (Fig. S14). These results suggest that the enhanced T-bet in macrophages by IL-23 is involved in the IFN-γ but not IL-17A, IL-17F and IL-22 expression.

### The roles of M(IL-23) macrophages in a psoriasis model

**Figure 6:**
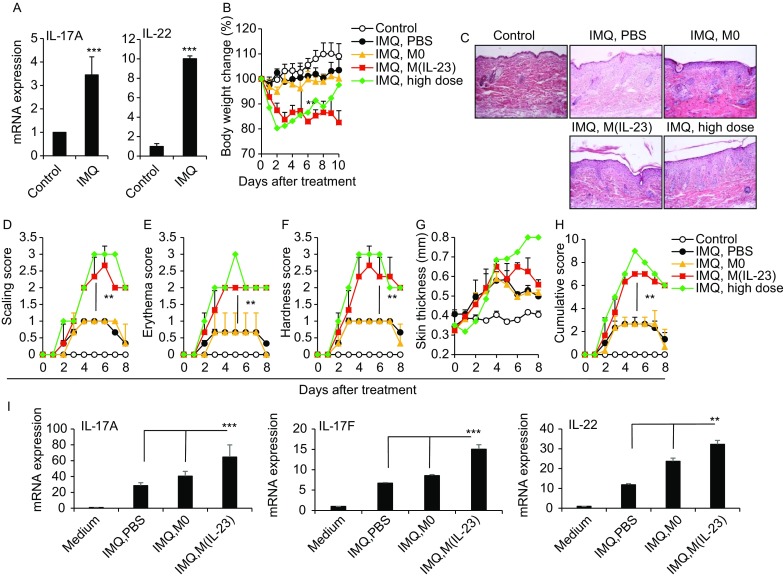
**M(IL-23) promoted the pathogenesis in imiquimod-induced psoriasis mice**. (A) mRNA expression of IL-17A and IL-22 in the sorted F4/80^+^ macrophages of skin tissue. (B) We used a suboptimal dose of IMQ (35 mg/day), and a standard dose of IMQ (70 mg/day) was used as a positive control (IMQ, high dose). The IMQ (35 mg/day)-treated mice were transferred with PBS, 1 × 10^6^ M0 or M(IL-23) macrophages. Body weight loss was shown. (C) H&E staining of the skin tissues. Scaling score (D), erythema score (E), hardness score (F), skin thickness (G) and cumulative score (H) of mice. (I) mRNA expression of IL-17A, IL-17F, and IL-22 in skin tissues. Data were shown as mean ± SD (*n* = 8). **P < 0.01, ***P < 0.001 for comparisons between the indicated groups

IL-23 and IL-17 are crucial in the pathogenesis of psoriasis, EAE, CIA, IBD (Cua et al., 2003; Murphy et al., 2003; Wilson et al., 2007; Tonel et al., 2010) and LAD1 (Moutsopoulos et al., 2017). We employed an imiquimod (IMQ)-induced murine model of psoriasis, in which Th17 cytokines like IL-23 and IL-17 were highly involved (van der Fits et al., 2009; Imai et al., 2015). CD11b^+^F4/80^+^macrophages sorted from skin tissue (Fig. S15) with IMQ-induced psoriasis-like dermatitis expressed high levels of IL-17A and IL-22 molecules (Fig. 6A). In order to investigate whether the induced M(IL-23) cells could promote the pathogenesis in the IMQ-induced psoriasis mouse model, we used a suboptimal dose of IMQ (about 35 mg per mouse) to induce a weak psoriasis-like dermatitis. A suboptimal dose of IMQ treatment caused a weak but observable dermatitis, while the treatment with a standard dose of IMQ (about 70 mg per mouse) induced a typical clinical and pathological alteration of psoriasis-like dermatitis as indicated by the body weight loss, scores of skin scaling, erythema, hardness and thickness, as well as skin pathological changes (Fig. 6B–H). Adoptive transfer of IL-23-induced M(IL-23) macrophages into recipient mice with suboptimal IMQ treatment significantly enhanced the severity of dermatitis to a degree caused by the treatment of a high dose of IMQ, as evidenced by the alterations of body weight, skin scaling, erythema, hardness, thickness, and pathological changes, whereas adoptive transfer of resting macrophages failed to do so (*P* < 0.01, Fig. 6B–H). In parallel to the pathogenesis, the IL-17A, IL-17F, and IL-22 mRNA expression in skin tissues were significantly increased by adoptive transfer of M(IL-23) macrophages (*P* < 0.001, Fig. 6I). Thus, M(IL-23) macrophages have the ability to promote the pathogenesis in a mouse model with psoriasis-like dermatitis.

## Discussion

Macrophage polarization is determined by genetic and environmental factors. Macrophage polarizations play a critical role in mastering the amplitudes and types of host immunity. In the present study, we identified a previously unappreciated macrophage polarization, that is, M(IL-23) macrophage subpopulation with the following characteristics and supporting evidences: 1) IL-23-treated resting macrophages display distinct gene expression profiling than M1 and M2 macrophages; 2) IL-23-treated resting macrophages selectively produce IL-17A, IL-17F, IL-22 and IFN-γ, but not M1 and M2-related cytokines and molecules including TNF-ɑ, IL-12, iNOS, Arg1, YM1, and FIZZ1; 3) Resting macrophages are susceptible to M(IL-23) induction, while polarized M1 and M2 macrophages are highly resistant to IL-23 treatment. 4) IL-23-treated resting macrophages present in psoriasis-like dermatitis and promote pathogenesis; and 5) IL-23-induced IL-17A, IL-17F and IL-22 expression in macrophages is dependent on STAT3-RORγT pathway, while the expression of IFN-γ in IL-23-treated macrophages was likely mediated by T-bet pathway. Therefore, IL-23-treated macrophages display distinct phenotype and cytokine production compared with M1 and M2 macrophages.

It is reported that IL-23 significantly contributes to inflammatory disease risk in humans (Duerr et al., 2006; Genetic Analysis of Psoriasis et al., 2010). Mice deficient in IL-23 but not IL-12 are resistant to experimental immune-mediated disease like EAE, RA, and IBD (Cua et al., 2003; Murphy et al., 2003). The promotion of Th17 subset is highly recognized to be the key player to mediate the critical role of IL-23 in inflammatory diseases and infection-induced pathological consequences like Lyme disease and toxoplasma encephalitis (Weaver et al., 2013). Our present study shows that IL-23 acts directly on macrophages to induce IL-17A, IL-17F, IL-22 and IFN-γ productions which likely promote the severity of psoriasis-like dermatitis in mice. The ability of IL-23 to induce IL-17 production in macrophages is consistent with the recent observations showing that IL-17 production by macrophages contributes to allergic asthma and that IL-23 protection against plasmodium berghei infection in mice is partially dependent on IL-17 from macrophages (Song et al., 2008; Ishida et al., 2013). The significance of M(IL-23) macrophage polarization in Th17 cytokines-related inflammatory diseases requires to be clarified.

IL-23, an IL-12 cytokine family member, is a heterodimeric molecule composed of p40 and p19 subunits (Langrish et al., 2005). The known biological roles and the pro-inflammatory activities of IL-23 in inflammation and autoimmune diseases include but not limit to the induction of Th17-induced secretion of IL-17 and suppression of CD4^+^CD25^+^ regulatory T cells (Iwakura and Ishigame, 2006; Izcue et al., 2008). IL-23 signals through IL-23R and IL-12Rβ1 to activate JAK and predominantly the phosphorylation and activation of STAT3 (Oppmann et al., 2000), which acts to promote transcription of Il23r and Rorc (encoding RORγ), establishing a positive feedback loop and stabilizing expression of genes encoding pro-inflammatory effector molecules including Il17a, Il17f, Il22 and Csf2 (Parham et al., 2002; Codarri et al., 2011). In macrophages, IL-23 uses the classical STAT3-RORγT pathway to induce Th17 cytokines gene expression profile. On the other hand, IFN-γ is characteristically produced by NK, T and NKT cells. It is reported that monocytes/macrophages can express IFN-γ by IL-12/IL-18 and LPS/ATP stimulations, respectively (Raices et al., 2008). In the present study, LPS + IFN-γ and IL-4 failed to induce detectable IFN-γ expression in resting macrophages, but IL-23 drove resting macrophages to express high levels of IFN-γ via T-bet pathway.

In summary, we identified a unique macrophage subpopulation M(IL-23) induced by IL-23 with a distinct gene expression profile in contrast to M1 and M2 macrophages. Importantly, IL-23-induced M(IL-23) macrophage polarization is closely involved in the pathogenesis in an IMQ-induced psoriasis mouse model. The physiological function of M(IL-23) macrophages in tissue repair and remodeling, as well as the role of M(IL-23) macrophages in pathogenesis caused by infections, tumors and graft rejection need to be explored in the future.

## Materials and methods

### Animals and reagents

C57BL/6(B6) mice were purchased from Beijing University Experimental Animal Center. ROR-γt knock-out (KO) mice (B6.129P2(Cg)-*Rorc*^tm2Litt^/J; JAX; Stock No.: 007572) were purchased from The Jackson Laboratory. All mice were maintained in a specific pathogen-free facility. All experimental manipulations were undertaken in accordance with the Institutional Guidelines for the Care and Use of Laboratory Animals, Institute of Zoology.

Recombinant mouse cytokines were purchased from PeproTech (Rocky Hill, NJ). RmIL-21 and IL-23 were obtained from R&D Systems (Minneapolis, MN). Bacterial lipopolysaccharide (LPS; *E*. *coli* 055:B5) was purchased from Sigma-Aldric (St Louis, MO). Selective STAT3 inhibitor (NSC74859; 4655/10) were obtained from R&D Systems and RORγT inverse agonist (SR2211; 557353) were from The Merck Group (Darmstadt, Germany). The reagents were used at the indicated or following concentrations based on our previous studies (Hou et al., 2013; Hou et al., 2014): recombinant mouse IL-1β (100 ng/mL), IL-2 (100 U/mL), IL-4 (1000 U/mL), IL-6 (20 ng/mL), IL-10 (20 ng/mL), IL-12 (10 ng/mL), IL-13 (20 ng/mL), IL-17A (100 ng/mL), IL-21 (100 ng/mL), IL-23 (100 ng/mL), IL-33 (100 ng/mL), TNF-α (100 ng/mL), IFN-γ (50 ng/mL) TGF-β1 (5 ng/mL); and recombinant human IL-23 (100 ng/mL), M-CSF (10 ng/mL); LPS (500 ng/mL), NSC74859 (100 μmol/L), SR2211 (10 μmol/L).

Anti-mF4/80-PE-Cy5, anti-mCD11b-PE-Cy5, anti-mTNF-α-FITC and anti-mCXCR5-PE were purchased from BD Biosciences Pharmingen (San Diego, CA, US). Anti-mIL-17A-PE was purchased from Biolegend (San Diego, CA, US). Anti-mIL-23R-AF488 mAb was purchased from R&D Systems. The primary antibodies against p-STAT3 (Tyr705), p-STAT3 (Ser727), STAT3, IRF4 and BATF were purchased from Cell Signaling Technology (Beverly, MA, US). The primary antibodies against RORγT were from Millipore Biotechnology (Billerica, MA, US).

### Cell isolation

Primary mouse peritoneal macrophages were obtained from B6 mice as described previously (Zhu et al., 2014). The purity of macrophages was more than 90% of CD11b^+^F4/80^+^macrophages as analyzed by flow cytometry (Yang et al., 2016). For real-time PCR and ELISA assays, CD11b^+^F4/80^+^ macrophages were further sorted by a MoFlo XDP High Speed Cell Sorter (Beckman Coulter).

### Microarray hybridization and data analysis

Total RNA was amplified and labeled by Low Input Quick Amp Labeling Kit, One-Color (Cat#5190-2305, Agilent technologies, Santa Clara, CA, US). Differentially expressed genes were defined as genes with at least 2 fold variance of expression levels in M1, M2 and M(IL-23) polarized macrophages compared to M0 macrophages.

### Immunofluorescent staining

Cells were cultured on coverslips for the indicated time and then fixed in 4% paraformaldehyde for 10 min and stored in PBS at 4°C (Hou et al., 2014). Cells were permeabilized and blocked and then were incubated with the indicated mAbs (1:100 dilution) overnight at 4°C. Following PBS washes, the secondary Ab was applied for 1 h and HOECHST33342 (2 μg/mL) for 10 min. Photomicrographs were taken using an LSM510META Laser Scanning Microscope (Zeiss, Germany).

### Western blot

It was performed as described (Sun et al., 2012). Protein bands were visualized by adding HRP membrane substrate (Millipore) and then scanned using the Tanon 1600R Gel Image System (Tanon Co., Ltd., Shanghai, China). GAPDH mAb (Proteintech Group, Inc) was used to normalize for the amount of loaded protein.

### ELISA

IL-17A, IL-17F, IL-22 and IFN-γ ELISA assays were performed following the manufacturer’s instructions (Biolegend).

### Quantitative PCR

Real-time PCR was performed using multiple kits (SYBR Premix Ex TaqTM, DRR041A, Takara Bio) on CFX96 (Bio-Rad) (Li et al., 2017). The primers are listed in Table S1. Housekeeping gene hypoxanthine phosphoribosyl transferase (HPRT) was used as an internal control.

### IMQ-induced psoriasis

The day that psoriasis was first induced by imiquimod (IMQ) was defined as day 0. On days −1 and 1, B6 mice were injected i.v. with 1 × 10^6^ IL-23-induced macrophages which were treated with IL-23 for 72 h *in vitro* as described above. B6 mice were used to induce psoriasis by IMQ as described previously (van der Fits et al., 2009). Mice were evaluated daily. Back redness (erythema), presence of scales (scaling), and hardness of the skin were scored using a semiquantitative scoring system from 0 to 4 based on their external physical appearance: 0 = no skin abnormalities, 1 = slight, 2 = moderate, 3 = marked, and 4 = severe. In addition, mice were weighed, and dorsal skin thickening was assessed by measuring double-skin fold thickness using a digital micrometer (Mitutoyo). At the end of the experiment, back skin samples were fixed in 4% formaldehyde and stained with H&E. Parakeratosis, acanthosis and leukocyte infiltration were assessed to evaluate scores in a blinded way. Scores from 0 to 2 were given, as follows: 0 = no abnormalities; 1 = psoriasis-like dermatitis: epidermal acanthosis, reduction of granulose layer, and hyperkeratosis with modest leukocyte infiltration; 2 = psoriasis-like dermatitis: higher epidermal acanthosis, absence of granulose layer, and higher hyperkeratosis with leukocyte infiltration enriched in neutrophils.

### Intracellular cytokine staining

Macrophages were treated with GolgiPlug (BD Pharmingen) for the last 6 h of incubation (Zhu et al., 2014). Cells were fixed and permeabilized with fixation and permeabilization solution (BD; 553722) and Perm/Wash buffer (BD; 554723). Cells were analyzed for the intracellular production of cytokines by staining with anti-mTNF-ɑ-FITC, anti-mIFN-γ-PE, or anti-mIL-17A-PE, respectively. The cells were then detected by a flow cytometry.

### Statistical analysis

Data are presented as mean ± SD. Student’s unpaired *t* test for comparison of means was used. For multiple group comparison, significant difference was calculated using the non-parametric Mann-Whitney U test. A *P* value less than 0.05 was considered significant.

## Electronic supplementary material

Below is the link to the electronic supplementary material.

Supplementary material 1 (PDF 879 kb)
