# Supplementary material for: IL-23-induced macrophage polarization and its pathological roles in mice with imiquimod-induced psoriasis
Source: Protein Cell. 2018 Mar 5;9(12):1027–38. doi: 10.1007/s13238-018-0505-z (PMC6251802; doi:10.1007/s13238-018-0505-z)
Supplement: Supplementary file 1 — Supplementary material 1 (PDF 879 kb) [file 13238_2018_505_MOESM1_ESM.pdf]

IL-23-induced macrophage polarization and its pathological roles in mice with imiquimod-induced psoriasis

Yuzhu Hou[*,¶], Linnan Zhu[*,¶], Hongling Tian[*, ,¶], Hai-Xi Sun[*], Jun Lu , Ruoyu Wang[,#], Lianfeng Zhang[§ ,#], Yong Zhao[*,#]

•State Key Laboratory of Membrane Biology, Institute of Zoology, Chinese Academy of Sciences; Department of Oncology, the Affiliated Zhongshan Hospital of Dalian University; Hepatology and Cancer Biotherapy Ward, Beijing YouAn Hospital, Capital Medical University; [§] Key Laboratory of Human Diseases Comparative Medicine, Ministry of Health; Institute of Laboratory Animal Science, Chinese Academy of Medical Sciences and Peking Union Medical College, China

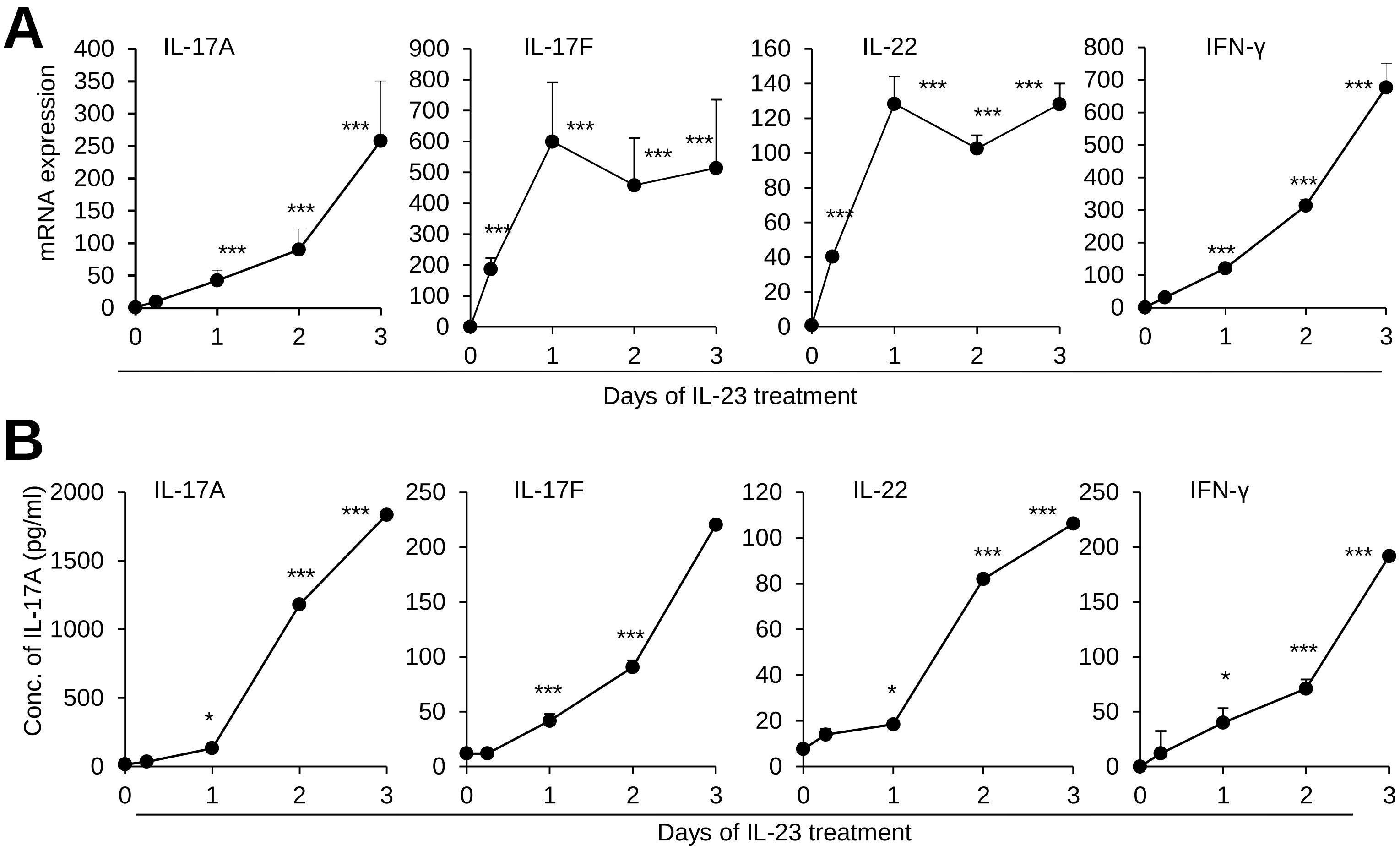

Suppl. Fig. 1. Cytokine expression in IL-23-treated macrophages.

A, mRNA expression of IL-17A, IL-17F, IL-22 and IFN-γ in mouse peritoneal macrophages (PEMs) treated with 100ng/ml IL-23 for indicated time points were detected by real-time PCR. **B**, Concentrations of IL-17A, IL-17F, IL-22 and IFN-γ in culture media of PEMs treated with 100ng/ml IL-23 for indicated time were detected by ELISA. Data shown as mean±SD (n=3), which represent one of at least three independent experiments with similar results. *P<0.05, **P<0.01, ***P<0.001 compared with the control.

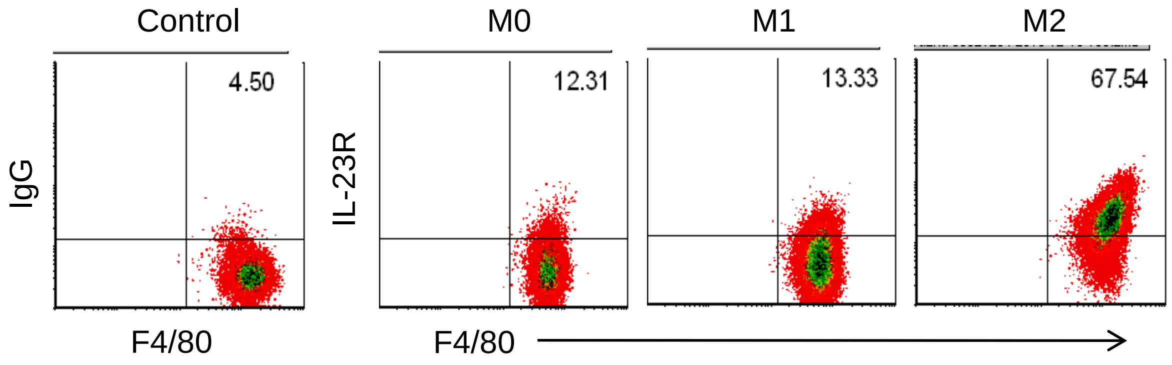

Suppl. Fig. 2. IL-23R expression on macrophages.
The IL-23R expression on M0，M1 and M2 macrophages of B6 mice were detected by multiple-colors flow cytometry. M0: the isolated peritoneal macrophages were untreated for 48 hrs in vitro; M1: the isolated peritoneal macrophages were treated with IFN-γ for 26 hrs and LPS stimulation for the last 6 hrs; M2: IL-4 treatment for 48 hrs in vitro. IgG is for negative control.

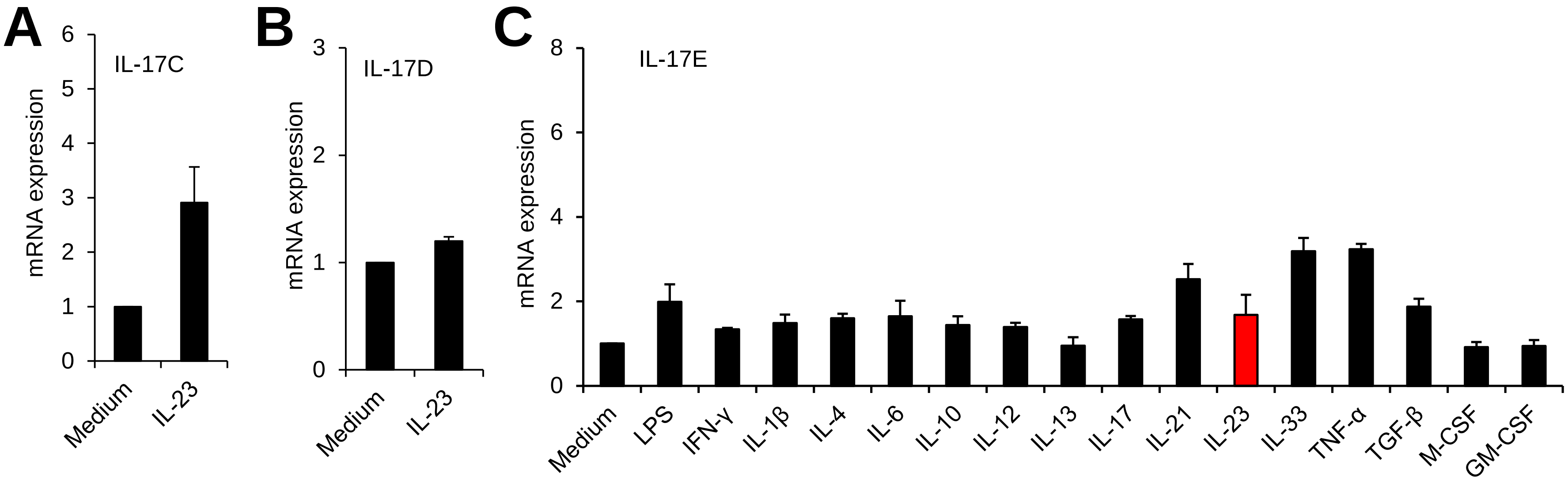

Suppl. Fig. 3. IL-17C, IL-17D and IL-17E expressions in IL-23-treated macrophages.
The mRNA expression of IL-17C (**A**) and IL-17D (**B**) in mouse PEMs treated with 100ng/ml IL-23 for 48 hrs were detected by real-time PCR. **C**, mRNA expression of IL-17E in PEMs treated with indicated cytokines and LPS for 48 hrs were detected by real-time PCR. Data are shown as mean±SD (n=3), which represent one of at least three independent experiments with similar results.

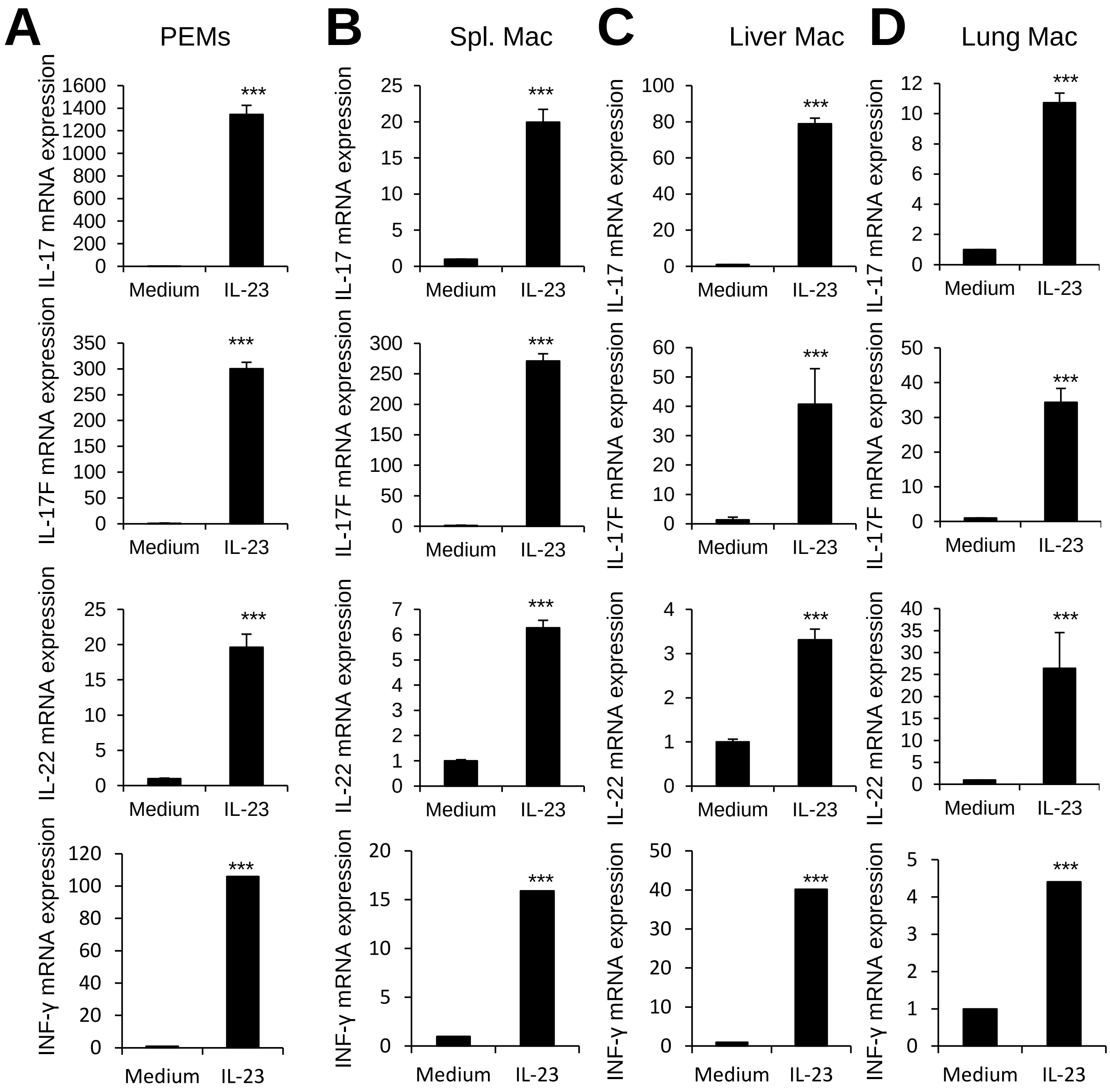

Suppl. Fig. 4. IL-17A, IL-17F, IL-22 and IFN-γ expressions in IL-23-treated tissue residence macrophages. The mRNA expression of IL-17A, IL-17F, IL-22 and IFN-γ in PEMs (**A**), the sorted F4/80+ macrophages from mouse spleen (**B**), the sorted F4/80+ macrophages from mouse liver (**C**), the sorted F4/80+ macrophages from mouse lung (**D**) treated with 100ng/ml IL-23 for 48 hrs were detected by real-time PCR. Data are shown as mean±SD (n=3), which represent one of two independent experiments with similar results. *P<0.05, **P<0.01, ***P<0.001 compared with the control.

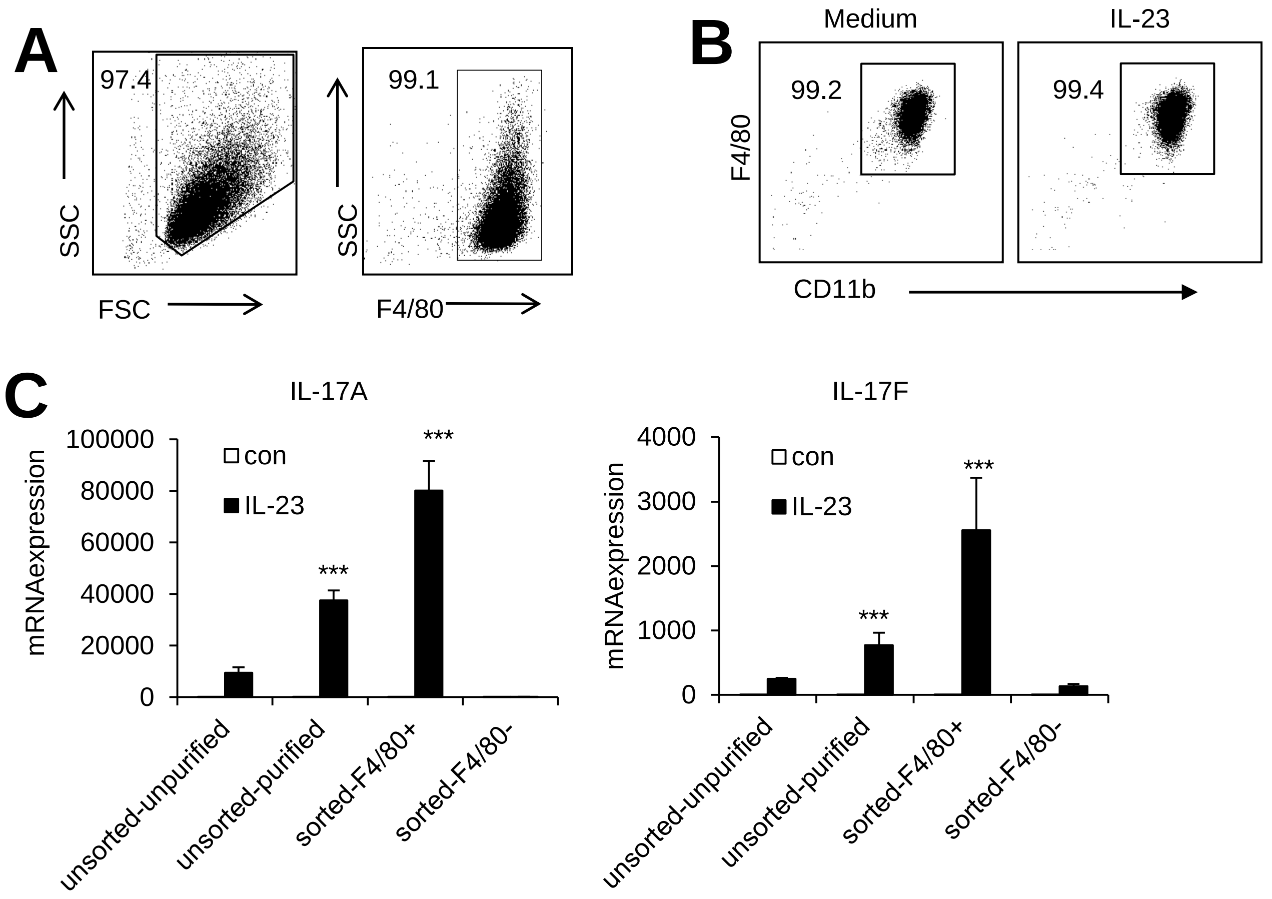

Suppl. Fig. 5. The Th17 cytokines expression in highly purified F4/80+ mouse peritoneal macrophages.
A, The percentage of sorted F4/80+ macrophages from mouse peritoneal cells by using flow cytometry was more than 99%. **B**, The percentage of sorted F4/80+ macrophages after the sorted mouse peritoneal cells were cultured with IL-23 for 48hrs was more than 99% as determined by flow cytometry . **C**, The mRNA expression of IL-17A and IL-17F in the sorted F4/80^{+} macrophages treated with 100ng/ml IL-23 for 48 hrs were detected by real-time PCR. Data are shown as mean±SD (n=3). *P<0.05, **P<0.01, ***P<0.001 compared with the control.

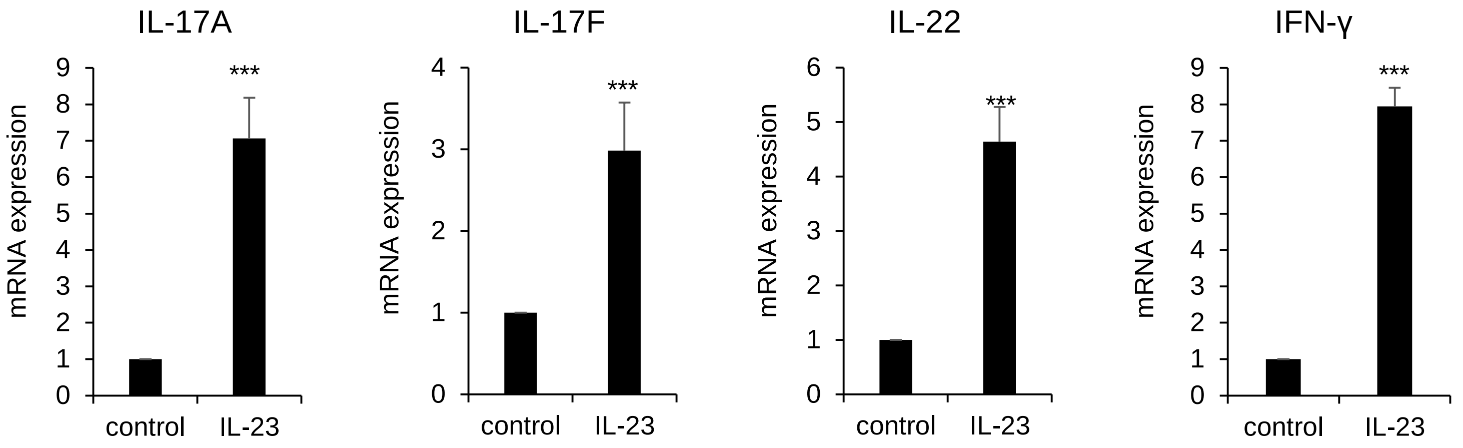

Suppl. Fig. 6. IL-17A, IL-17F, IL-22 and IFN-γ expressions in IL-23-treated bone marrow-derived macrophages.

Macrophages were derived from bone marrow cells in vitro. The mRNA expression of IL-17A, IL-17F, IL-22 and IFN-γ in bone marrow-derived macrophages treated with 100ng/ml IL-23 for 48 hrs were detected by real-time PCR. Data are shown as mean±SD (n=3), which represent one of two independent experiments with similar results. ***P<0.001 compared with the control.

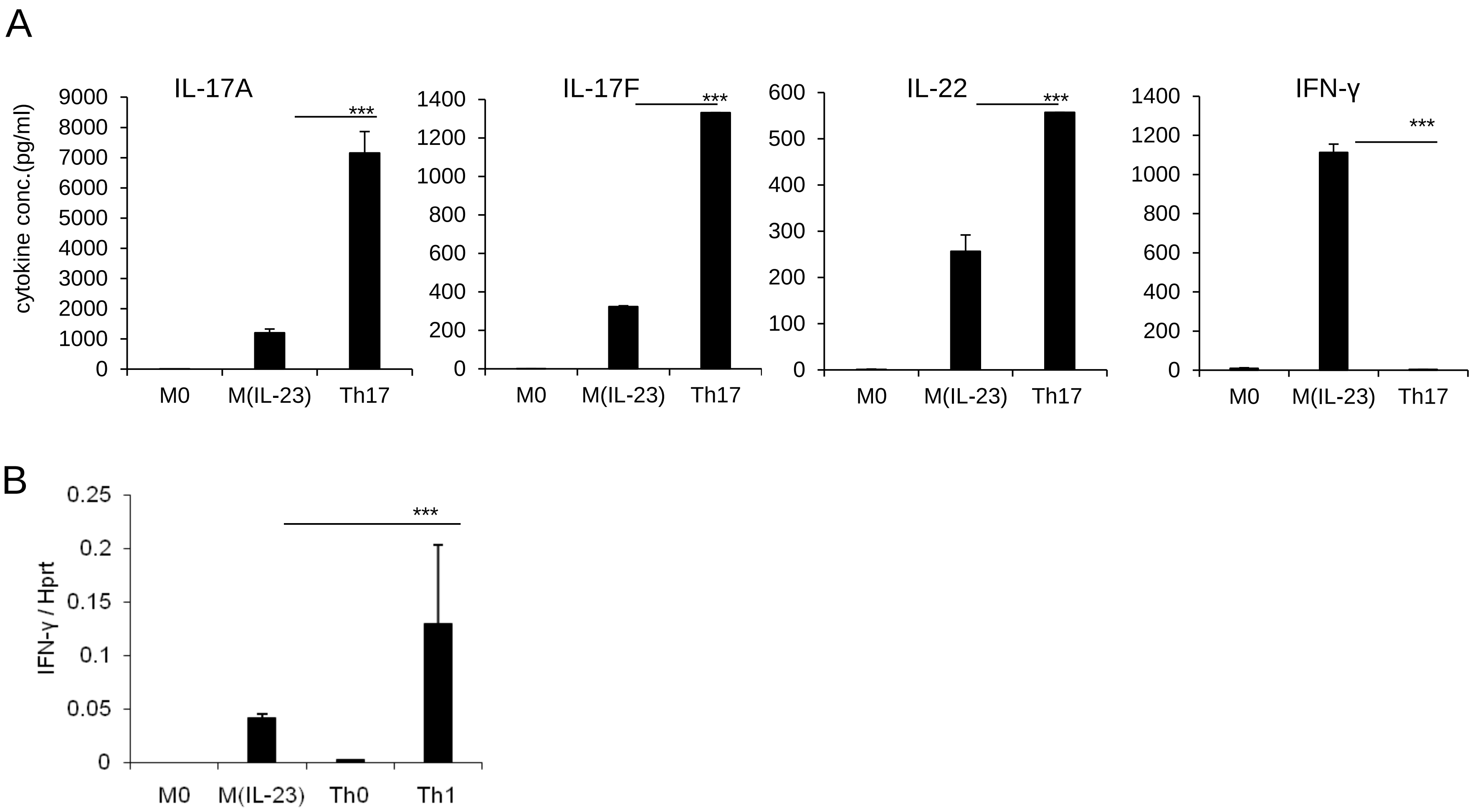

Suppl. Fig. 7. IL-17A, IL-17F, IL-22 and IFN-γ expressions in M(IL-23), Th17 and Th1.

A. Cell culture supernatant were collected from the same number M(IL-23) and Th17 cells respectively, and IL-17A, IL-17F, IL-22 and IFN-γ expression were determined by ELISA. Th17 was used as a positive control for IL-17A, IL-17F, IL-22 expression. **B.** mRNA were extracted from M(IL-23) and Th1 cells, and IFN-γ were detected by real-time PCR. Data are shown as mean±SD (n=3), which represent one of three independent experiments with similar results. ***P<0.001 compared with the control.

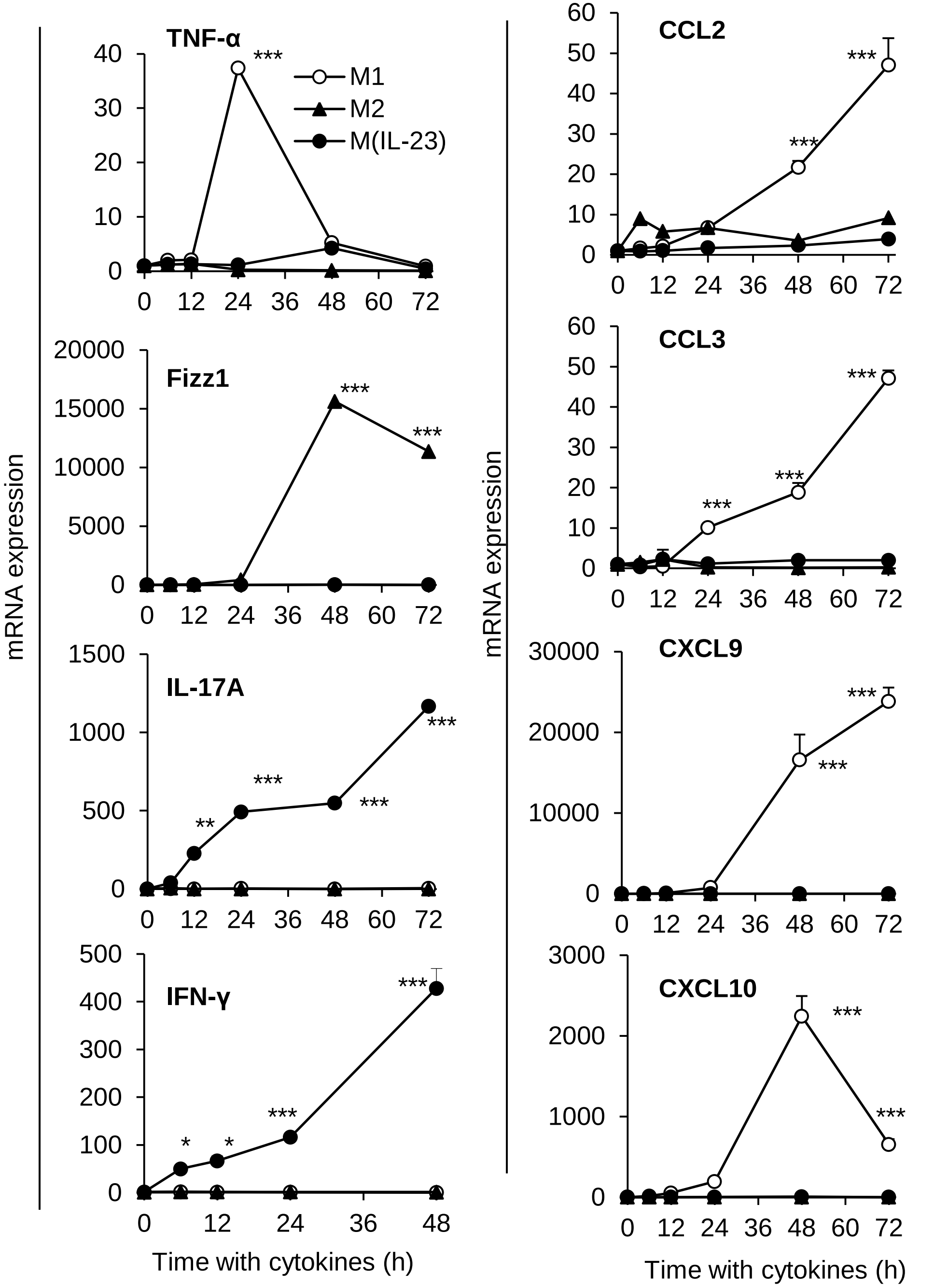

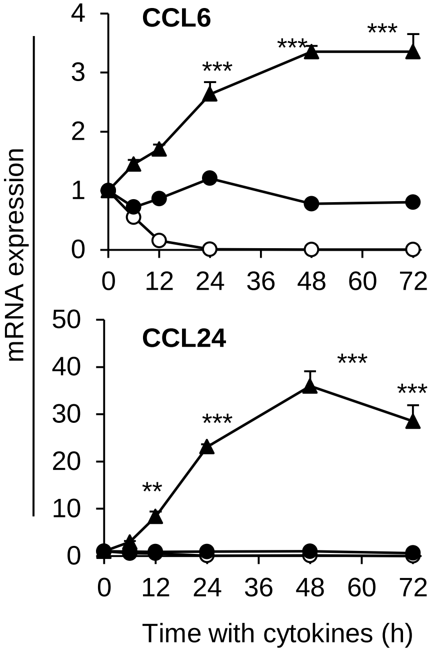

Suppl. Fig. 8. The expressions of marker molecules of M1, M2, and M(IL-23) macrophages.

The expression of M1 marker molecules (including TNF-α, CCL2, CCL3, CXCL9 and CXCL10), M2 marker molecules (including Fizz1, CCL6 and CCL24) and M(IL-23) maker molecules (including IL-17A and IFN-γ) at indicated culture time points were detected by real-time PCR. Data are shown as mean±SD (n=3), which represent one of at least three independent experiments with similar results. *P<0.05, **P<0.01, ***P<0.001 compared with the control.

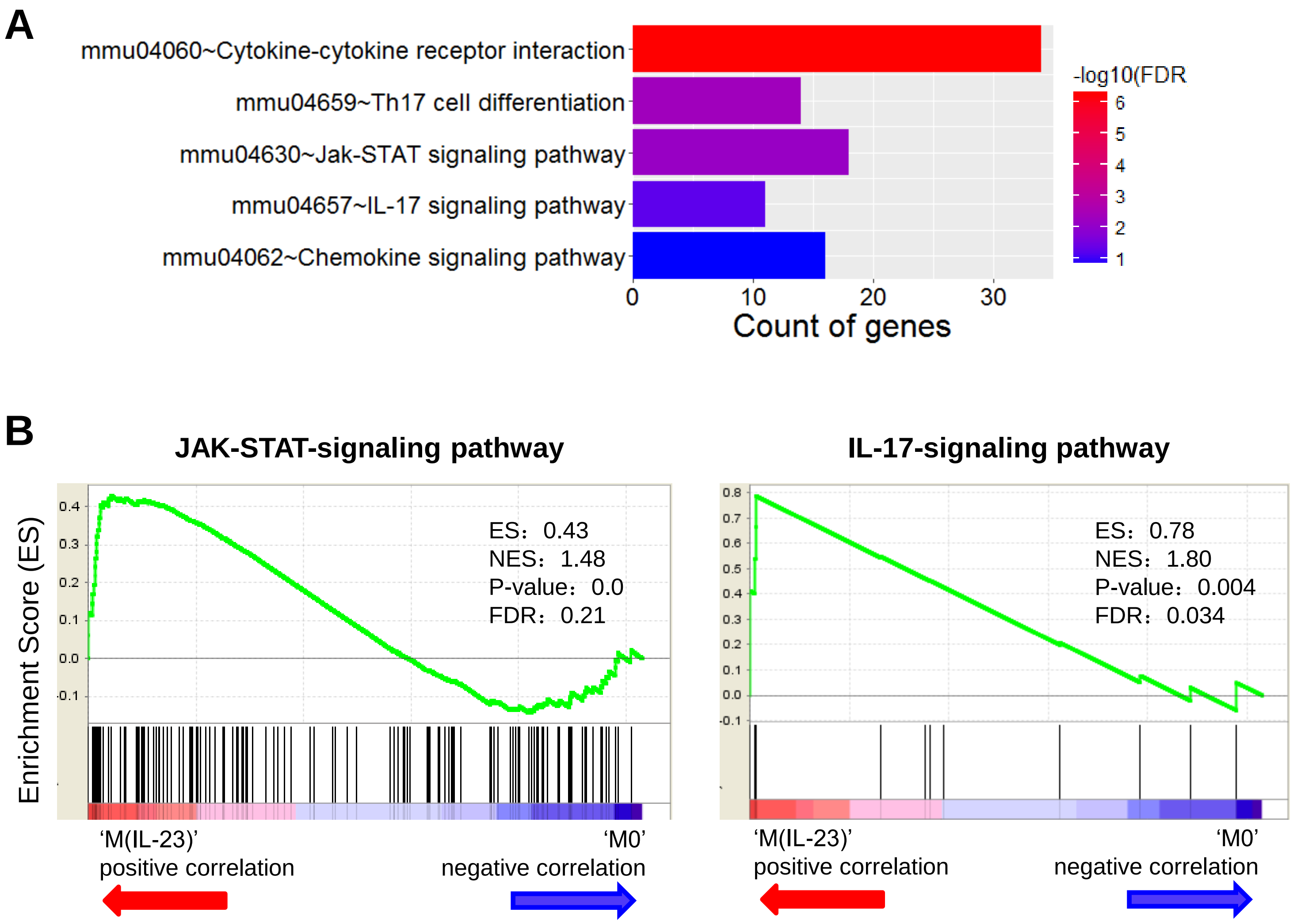

Suppl. Fig. 9. Signaling pathway analysis based on the gene expression data.
A. Signaling pathways involved in the activation of macrophages by IL-23. **B.** Gene set enrichment analysis (GSEA). Enrichment scores are shown for 132 genes of JAK-STAT3 signaling pathway (right) and 12 genes of IL-17-signaling pathway (left). Genes are ranked according to their expression changes induced by IL-23. ES, enrichment score; NES, normalized enrichment score; FDR, false discovery rate.

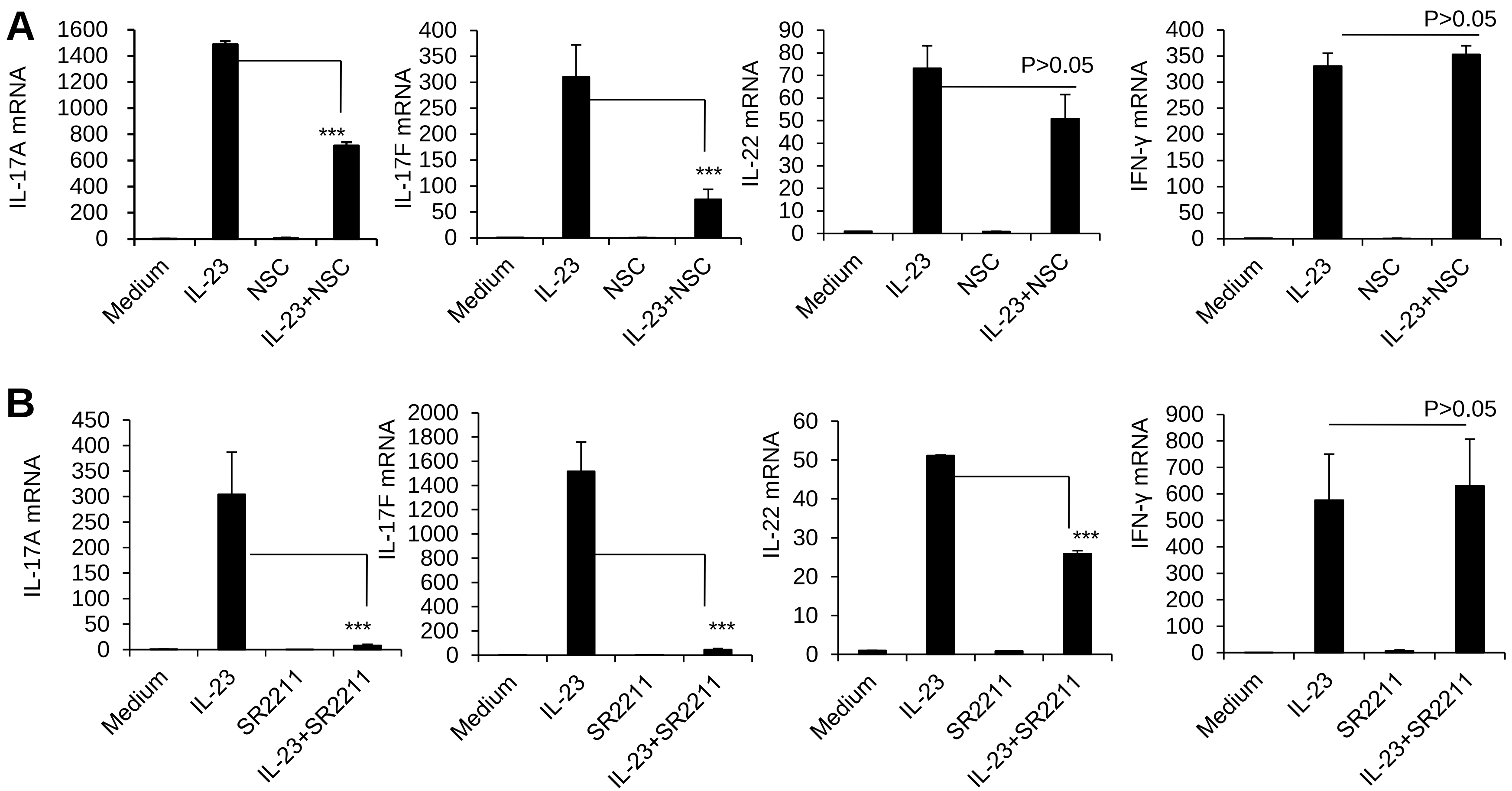

Suppl. Fig. 10. The role of STAT3 and RORγT in M(IL-23) induction by IL-23.

A, mRNA expression of IL-17A, IL-17F, IL-22 and IFN-γ in macrophages treated with or without IL-23 and/or STAT3 inhibitor NSC74859 for 48 hrs were determined by real-time PCR. **B**, mRNA expression of IL-17A, IL-17F, IL-22 and IFN-γ in macrophages treated with or without IL-23 and/or RORγT inhibitor SR2211 for 48 hrs were determined by real-time PCR. Data are shown as mean±SD (n=3), which represent one of at least three independent experiments with similar results. *P<0.05, **P<0.01, ***P<0.001 compared with the indicated groups.

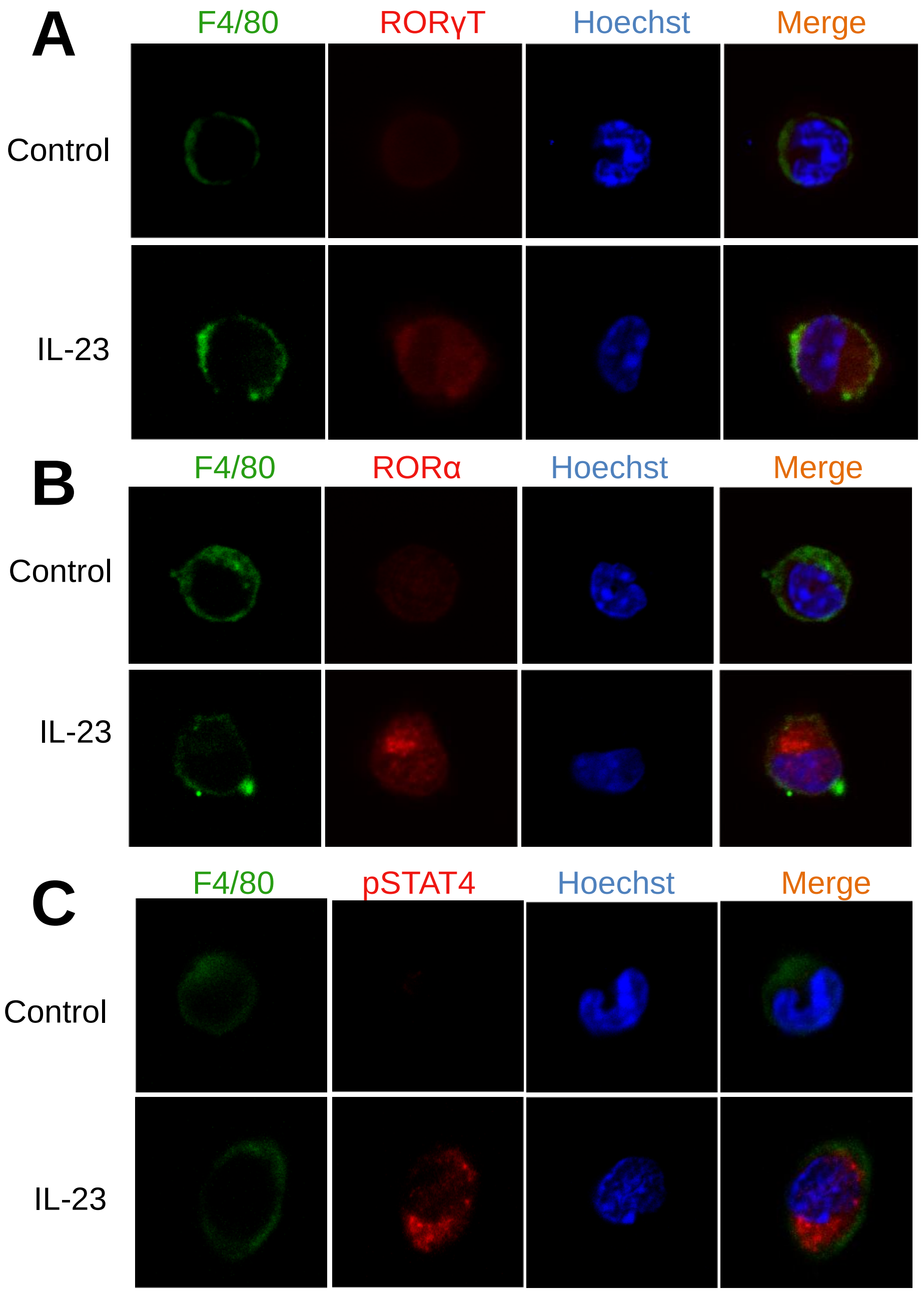

Suppl. Fig. 11. The expression of RORγT and RORα and phosphorylation state of STAT4 in M(IL-23) macrophages.
The expression and location of RORγT (**A**) and RORα (**B**) in F4/80+ macrophages treated with or without 100ng/ml IL23 for 48 hrs were determined using two-photon microscope. **C**, The phosphorylation state of STAT4 in F4/80+ macrophages treated with or without 100ng/ml IL23 for 48 hrs was determined using two-photon microscope.

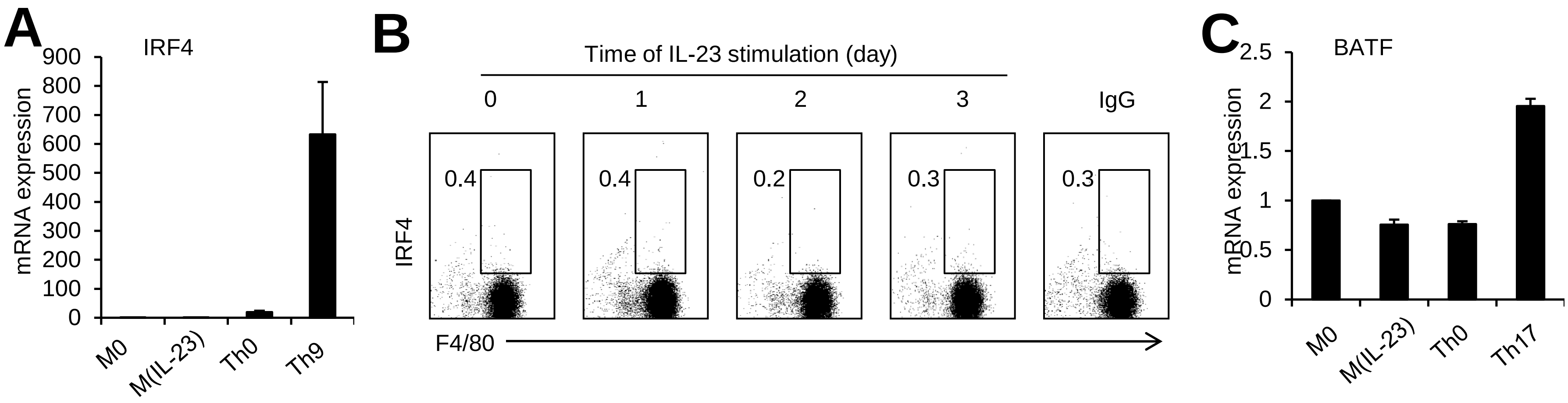

Suppl. Fig. 12. The expressions of IRF4 and BATF in M(IL-23) macrophages.
A, mRNA expression of IRF4 in macrophages treated with or without 100ng/ml IL23 for 48 hrs determined using real-time PCR. Th9 was used as a positive control for IRF4 expression. **B**, IRF4 expression in F4/80+ macrophages treated with IL-23 for indicated time was determined using flow cytometry. **C**, mRNA expression of BATF in macrophages treated with or without 100ng/ml IL-23 for 48 hrs was determined using real-time PCR. Th17 was used as a positive control for BATF expression. Data are shown as mean±SD (n=3), which represent one of at least three independent experiments with similar results.

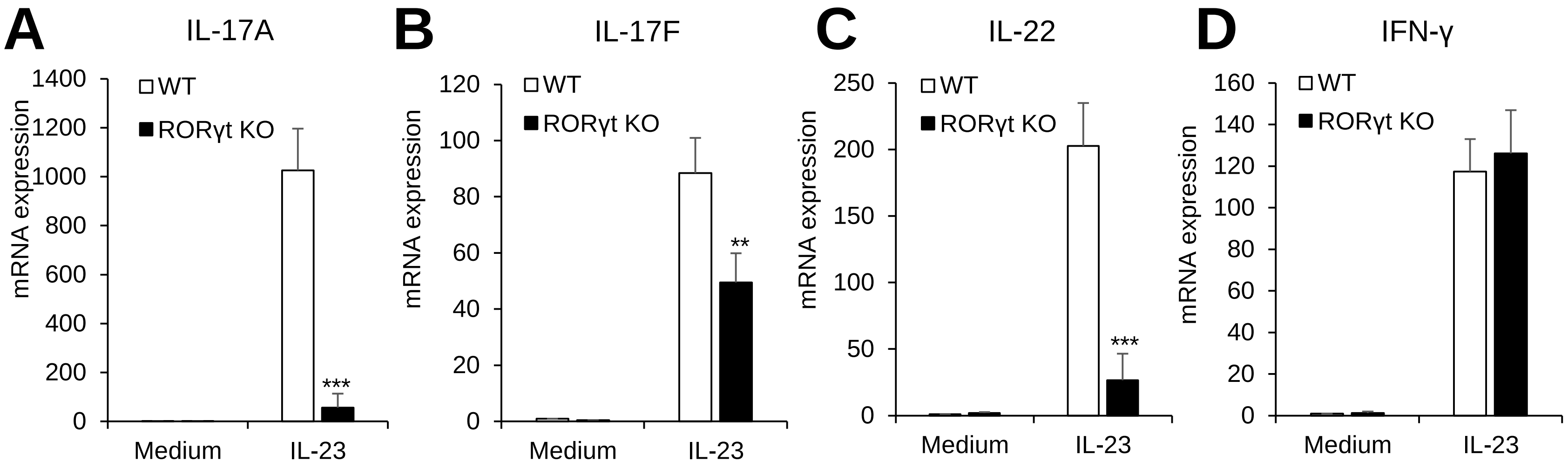

Suppl. Fig. 13. ROR-γt deficiency has significant effect on the expressions of IL-17A, IL-17F, and IL-22 in M(IL-23) macrophages.

The freshly isolated WT and ROR-γt KO CD11b^{+}F4/80^{+} PEMs were cultured with control medium and 100ng/ml IL-23 for 48 hrs. The mRNA expression analyzed by using real-time PCR. Data are shown as mean±SD (n=3), which represent one of at least three independent experiments with similar results. **P<0.01, ***P<0.001 compared with the WT control group.

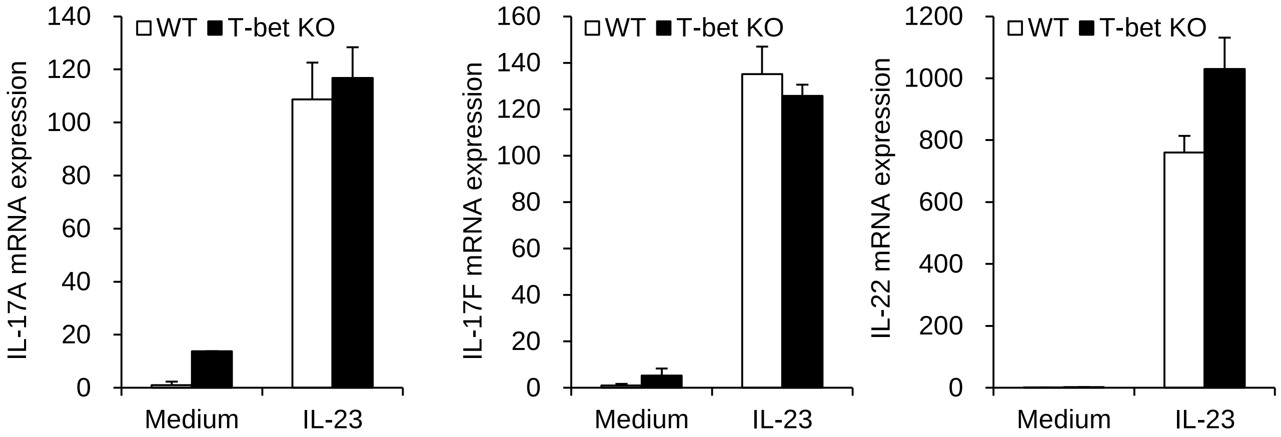

Suppl. Fig. 14. T-bet deficiency has no significant effect on the expressions of IL-17A, IL-17F, IL-22 in M(IL-23) macrophages.

WT and T-bet KO PEMs were cultured with control medium and 100ng/ml IL-23 for 48 hrs. The mRNA expressions analyzed by using real-time PCR. Data are shown as mean$\pm$SD (n=3), which represent one of at least three independent experiments with similar results.

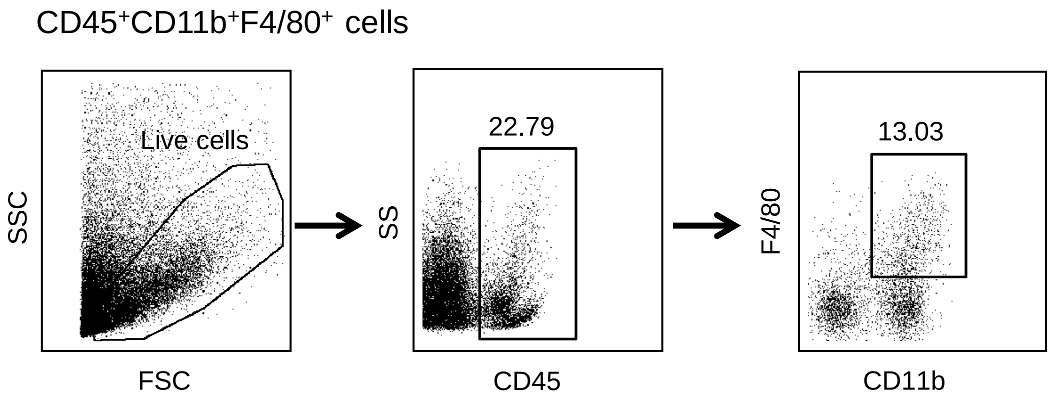

Suppl. Fig. 15. Gating strategy used to identify CD45$^+$CD11b$^+$F4/80$^+$ macrophages present in skin based on the surface markers.
Total cells from skin of B6 mice were processed and analyzed as described in Materials and Methods. Data shown are representative of >3 experiments.

Supplementary table 1. Primers used for real-time PCR analysis

Genes	Primer sequence (5'→3')	
IL-1β	Forward	TGGGAAACAACAGTGGTCAGG
	Reverse	CCATCAGAGGCAAGGAGGAA
IL-12β	Forward	CACGGCAGCAGAATAAATA
	Reverse	CTTGAGGGAGAAGTAGGAATG
IL-17A	Forward	CTCAGACTACCTCAACCGTTCC
	Reverse	ATGTGGTGGTCCAGCTTTCC
IL-17B	Forward	ATGGGGCTACAGCATCAACC
	Reverse	CTCACCATGCTACGGTCCTC
IL-17C	Forward	TGGAGATATCGCATCGACACA
	Reverse	CTGTCTCACGGCCTGTCTTG
IL-17D	Forward	GGCGCCCTTATTTACTTCGCA
	Reverse	AGCATCCAGACCAGTGTCCC
IL-17E	Forward	TTGGACAGGGACTTGAATCG
	Reverse	TCTGGTTGTGGTAAAGTGGG
IL-17F	Forward	CATACCCAGGAAGACATACTTAGAAG
	Reverse	AGTCCCAACATCAACAGTAGC
IL-21	Forward	CCAAACTCAAGCCATCAAACC
	Reverse	CTCATACGAATCACAGGAAGGG
IL-22	Forward	CTGAGAAATGCTTGCGTCTG
	Reverse	CGTTAGCTTCTCACTTTCCTTTAG
TNF-α	Forward	GAGTGACAAGCCTGTAGCC
	Reverse	CTCCTGGTATGAGATAGCAAA
IFN-γ	Forward	GAACTGGCAAAAGGATGGTGA
	Reverse	TGTGGGTTGTTGACCTCAAAC
CXCL2	Forward	GCCCAGACAGAAGTCATAGCC
	Reverse	CTCCTCCTTTCCAGGTCAGTTA
CXCL3	Forward	CCACCAACCACCAGGCTAC
	Reverse	GAGGCAAACTTCTTGACCATC
CXCL10	Forward	CGTCATTTTCTGCCTCATCC
	Reverse	GCAATGATCTCAACACGTGG
iNOS	Forward	CACCAAGCTGAACTTGAGCG
	Reverse	CGTGGCTTTGGGCTCCTC
Arginase1	Forward	CCAGAAGAATGGAAGAGTCAGTGT
	Reverse	GCAGATATGCAGGGAGTCACC
Fizz	Forward	CTGCCCTGCTGGGATGACT
	Reverse	CATCATATCAAAGCTGGGTTCTCC
Ym1	Forward	CAAGTTGAAGGCTCAGTGGCTC
	Reverse	CAAATCATTGTGTAAAGCTCCTCTC
RORα	Forward	TCCAAATCCCACCTGGAAAC
	Reverse	GAAGGTCTGCCACGTTATCTG
RORγT	Forward	GACCCACACCTCACAAATTGA

	Reverse	AGTAGGCCACATTACACTGCT
BATF	Forward	GCAGTGACTCCAGCTTCAG
	Reverse	TGTCGGCTTTCTGTGTCTG
IRF4	Forward	CTTTGAGGAATTGGTCGAGAGG
	Reverse	GAGAGCCATAAGGTGCTGTCA
T-bet	Forward	AGCAAGGACGGCGAATGTT
	Reverse	GGGTGGACATATAAGCGGTTC
HPRT	Forward	AGTACAGCCCCAAAATGGTTAAG
	Reverse	CTTAGGCTTTGTATTTGGCTTTTC
